# Épidémiologie de la lèpre dans le district sanitaire de Daoukro (Côte d'Ivoire) de 1999 à 2017

**DOI:** 10.48327/mtsi.v3i4.2023.294

**Published:** 2023-10-09

**Authors:** Ekissi Or sot TETCHI, Yao Eugène KONAN, Denise KPEBO, Mangou Christiane DJOMAN, Franck Kokora EKOU, Parfait Stéphane SABLE, Apolinaire YAPI, Odile TANO-AKE

**Affiliations:** 1 Département de santé publique et spécialités, UFR Sciences médicales, Université Félix Houphouët Boigny, Abidjan, Côte d'Ivoire; 2 Institut national de santé publique, Abidjan, Côte d'Ivoire; 3 Institut national d'hygiène publique, Abidjan, Côte d'Ivoire

**Keywords:** Lèpre, Épidémiologie, Daoukro, Côte d'Ivoire, Afrique subsaharienne, Leprosy, Epidemiology, Daoukro, Côte d'Ivoire, Sub-Saharan Africa

## Abstract

**Introduction:**

La lèpre est une réalité dans le district sanitaire de Daoukro malgré les actions du Programme national d’élimination.

**Objectif:**

L'objectif de cette étude est de décrire les aspects épidémiologiques et cliniques des nouveaux cas de lèpre dans le district sanitaire de Daoukro de 1999 à 2017.

**Méthode:**

Étude descriptive portant sur les patients lépreux reçus au service de dermatoléprologie de 1999 à 2017.

**Résultats:**

De 1999 à 2017, l'incidence annuelle de la lèpre fluctuait de 4,4 à 0 pour 100 000 avec un maximum de 14,2 pour 100 000 en 2003. À partir de 2004, elle est passée à moins de 1 cas pour 10 000 habitants (recommandations de l'OMS) pour se maintenir à 0 cas en 2016 et 2017. Avec un âge moyen de 36,8 ans (σ = 20), la majorité des cas étaient sans instruction et vivaient en milieu rural. Les sujets de sexe féminin et les enfants de moins de 15 ans représentaient respectivement 53% et 16% des cas. Sur le plan clinique, les signes cutanés prédominaient chez les patients. La forme multibacillaire représentait 82%. Près de 1 malade sur 4 présentait une invalidité de degré 2 (24%). Tous les patients reçus ont été mis sous traitement (polychimiothérapie). Parmi eux, 83,8% ont guéri, 0,5% étaient non guéris. Par ailleurs, les modalités évolutives n’étaient pas précisées chez 29 patients soit 15,7%. Parmi les patients déclarés guéris, 26% présentaient des séquelles.

**Conclusion:**

Les actions de lutte contre la lèpre doivent être renforcées en vue de maintenir les acquis dans ce district non endémique.

## Abstract (en)

**Introduction:**

Leprosy is a real problem in the Daoukro health district despite the actions of the National programme for elimination.

**Objective:**

To describe the epidemiological and clinical profile of new leprosy cases in the Daoukro health district from 1999 to 2017.

**Method:**

Descriptive survey including leprosy patients admitted to the dermato-leprology department from 1999 to 2017.

**Results:**

From 1999 to 2017, the incidence of leprosy fluctuated from 4.4 to 0 per 100,000 with a peak of 14.2 in 2003. In 2016 and 2017 no cases of leprosy were reported. The mean age was 36.8 years (SD=20). A majority of cases were uneducated and living in rural areas. Females and children under 15 years of age accounted for 53% and 16% of cases respectively. Clinically, cutaneous signs were predominant in the patients. The multibacillary form accounted for 82%. Nearly 1 out of 4 patients had a grade 2 disability (24%). All patients received treatment (multidrug therapy). Among them, 83.8% were cured, while 0.5% were not cured. In addition, regarding 29 patients (15.7%) details of how their disease progressed were not available. Among the patients declared cured, 26% had sequels.

**Conclusion:**

Leprosy control activities must be strengthened in order to maintain the achievements in this non-endemic district

## Introduction

La lèpre ou maladie de Hansen est une maladie infectieuse d’évolution chronique favorisée par la promiscuité et la pauvreté [2]. Elle sévit parmi les populations pauvres qu'elle appauvrit davantage [4, 10]. Sa gravité réside dans ses atteintes nerveuses fréquentes et les réactions lépreuses entraînant des difformités irréversibles chez la plupart des patients, notamment en cas de retard de diagnostic ou faute de traitement. Son évolution actuelle est rarement mortelle [6, 7, 15] tandis que le préjudice social et économique qu'elle entraîne est majeur. Les patients atteints de la lèpre continuent à souffrir d'ostracisme, bien que sa contagiosité réelle soit nettement moins importante que dans l'imaginaire collectif [15].

La lèpre est une maladie tropicale négligée (MTN) encore présente dans plus de 120 pays, et dont plus de 200 000 nouveaux cas sont notifiés chaque année. Grâce à une vaste campagne de détection des cas et d'instauration d'une polychimiothérapie en 1981 [13], l’élimination de la lèpre en tant que problème de santé publique a été accomplie à l’échelle mondiale en 2000 (conformément à l'objectif fixé dans la résolution WHA44.9 de l'Assemblée mondiale de la Santé), puis au niveau national dans la plupart des pays en 2010. La régression du nombre de nouveaux cas a été progressive, tant à l’échelle mondiale que dans les Régions de l'OMS [11]. Si les stratégies précédentes mettaient l'accent sur « l’élimination de la lèpre en tant que problème de santé publique », qui s'entend par une prévalence de moins de 1 cas sous traitement pour 10 000 habitants, la nouvelle stratégie privilégie l'interruption de la transmission et l'obtention de 0 cas autochtone [12]. Ainsi, la stratégie vise à motiver les pays à forte charge de morbidité lépreuse à accélérer leurs activités. L'Afrique subsaharienne reste la partie du continent africain la plus touchée par la lèpre [12]. En Côte d'Ivoire, elle continue de sévir à l’état endémique dans certains districts sanitaires malgré l'organisation de la lutte dans ces localités [8]. En 2021, le pays a déclaré moins de 500 cas de lèpre contre plus de 1000 cas en 2019. Dans le district sanitaire de Daoukro, bien que ne faisant pas partie des districts endémiques, des cas continuent d’être rapportés par les professionnels de santé [9]. La présente étude a pour objectif de décrire les aspects épidémiologiques et cliniques des nouveaux cas de lèpre dans le district sanitaire de Daoukro de 1999 à 2017.

## Méthodes

Type d’étude

Étude rétrospective des nouveaux cas de lèpre dépistés dans le district sanitaire de Daoukro de 1999 à 2017.

### Zone d’étude

Il s'agit du district sanitaire de Daoukro créé en 1996, situé dans la région du N'Zi-Comoé au centre-est de la Côte d'Ivoire, d'une superficie de 3745 km^2^. La population est estimée à 185 101 en 2018 [9], soit une densité de 38 habitants/km2. C'est une population essentiellement agricole. Depuis sa création, le district dispose d'un service de prise en charge de la lèpre, menant au niveau opérationnel les activités du Programme national d’élimination de la lèpre (PNEL). Il assure la coordination des activités de lutte contre la lèpre, sa surveillance épidémiologique, le dépistage précoce et le traitement adéquat des cas.

Depuis 2004, la prise en charge de la lèpre est assurée par un spécialiste en dermato-léprologie, coordonnateur-lèpre chargé d’éduquer la population, de dépister et faire traiter les cas et de rapporter les cas notifiés à la direction du district et au Programme de lutte contre la lèpre. Il travaille en collaboration avec le personnel des établissements sanitaires de premier contact (ESPC), l'hôpital de référence et les communautés qui lui réfèrent les cas suspects. Il assure ses missions sous l'autorité du médecin-chef du district sanitaire. Le diagnostic est essentiellement clinique.

### Collecte des données

Les données ont été extraites des dossiers médicaux à partir d'un questionnaire s'enquérant de divers paramètres:

- année de dépistage;
- caractéristiques sociodémographiques (âge, sexe, profession, nationalité, ethnie, niveau de scolarisation, situation matrimoniale, lieu de résidence);
- données cliniques (circonstances de découverte, les symptômes, les formes bacillaires, le degré d'invalidité);
- modalités évolutives (guérison, rechute, séquelles, décès).

### Traitement et analyse des données

La saisie des données a été faite avec l'application KoboCollect et le traitement des données avec le logiciel SPSS. Les variables quantitatives ont été exprimées sous forme de moyenne, de médiane et de quartile. Les variables qualitatives ont été présentées sous forme de proportion. L'incidence annuelle est obtenue en rapportant l'ensemble des nouveaux cas notifiés pour l'année sur la population totale de la même année. Un histogramme a permis de décrire l’évolution de la lèpre dans le district sanitaire de Daoukro.

### Considérations éthiques

Cette étude a obtenu l'accord de l’Équipe cadre du district sanitaire de Daoukro. La confidentialité des données provenant du service de la lèpre a été respectée car aucun nom de malade ni de village endémique n'a été mentionné.

## Résultats

**Figure 1:**
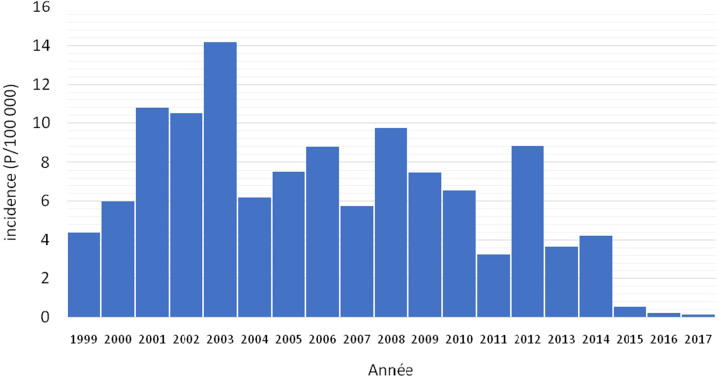
Incidence annuelle des cas de lèpre dans le district sanitaire de Daoukro de 1999 à 2017 Annual incidence of leprosy cases in the Daoukro health district from 1999 to 2017

Au total 185 nouveaux cas de lèpre ont été notifiés entre 1999 et 2017. La tendance générale est à la baisse de l'incidence malgré une augmentation entre 1999 et 2004, allant de 4,4 en 1999 pour atteindre 14,2 pour 100 000 habitants en 2003. À partir de 2004, cette incidence est passée à moins de 1 cas pour 10 000 habitants (recommandations de l'OMS) pour se maintenir à 0 cas en 2016 et 2017 (Fig. 1). Au niveau sociodémographique, 53,5% des patients étaient de sexe féminin et l’âge médian était de 32 ans (σ = 20). Aux premier et deuxième quartiles, les âges étaient respectivement de 20 et 51 ans. La majorité des patients résidaient en milieu rural (72%) et avaient au plus un niveau d’étude primaire (87%). Le Tableau I décrit les caractéristiques sociodémographiques des patients atteints de lèpre.

**Tableau I:** Répartition des 185 cas de lèpre selon les caractéristiques sociodémographiques Distribution of the 185 leprosy cases by socio-demographic characteristics

Variables	Femmes n	Hommes n	Ensemble n (%)
Tranches d’âge			
< 15	20	10	30 (16,2)
[15-30[	27	27	54 (29,2)
[30-45[	16	24	40 (21,6)
[45-60[	20	16	36 (19,5)
≥ 60	16	9	25 (13,5)
total	99	86	185 (100)
Milieu de résidence			
rural	78	56	134 (72,4)
urbain	21	30	51 (27,6)
Niveau d'instruction			
non scolarisé/aucun	70	53	123 (66,5)
primaire	21	17	38 (20,6)
secondaire	8	15	23 (12,4)
supérieur	0	1	1 (0,5)
Profession			
cultivateur, planteur, éleveur	0	26	26 (14,0)
ménagère	64	2	66 (35,7)
sans emploi	20	17	37 (20,0)
élève	10	9	19 (10,2)
autres (commerçant, guérisseur…)	5	31	36 (19,6)

La plupart des cas (61%) se sont présentés de façon spontanée au service de dermato-léprologie du district sanitaire. Les notifications faites par un agent de santé communautaire ont permis de dépister 32% des cas de lèpre. La quasi-totalité des patients (99%) présentaient au moins des signes cutanés à type de macules ou de nodule. La forme multibacillaire de la lèpre était prédominante (82%). Près de 1 patient lépreux sur 4 (24%) présentait une incapacité de degré 2 .Tous les cas avaient bénéficié de la polychimiothérapie telle que recommandée par l'OMS. Parmi eux, 83,8% ont guéri, 0,5% étaient non guéris. Par ailleurs, les modalités évolutives n’étaient pas précisées chez 29 patients soit 15,7%. Parmi les guéris, 26% présentaient des séquelles (Tableau II).

**Tableau II:** Répartition des 185 cas de lèpre selon les caractéristiques cliniques et évolutives Distribution of the 185 leprosy cases according to clinical and evolutive characteristics

Variables	Modalités	Effectif	Pourcentage
**Circonstances de découverte**	Examen de contact	3	1,6
	Examen de masse	3	1,6
	Examen sélectif	7	3,8
	Notification	59	31,9
	Présentation spontanée	113	61,1
**Signes cutanés**	Macule	148	80,0
	Mutilation ou ulcération	20	11,0
	Papule	8	4,3
	Nodules	6	3,3
	Plaques	3	1,6
**Forme bacillaire**	Multibacillaire	152	82,0
	Paucibacillaire	33	18,0
**Degré d'invalidité**	Degré 0	102	55,1
	Degré 1	38	20,5
	Degré 2	45	24,3
**Traitement**	PCTOMS*	185	100,0
	Pansement	20	10,8
	Anti-inflammatoires	15	8,1
**Modalités évolutives**	Guéri	155	83,8
	Non guéri	1	0,5
	Aucune information	29	15,7
**Type de guérison**	Sans séquelles	114	73,5
	Avec séquelles	41	26,5
**Rechute après guérison (n = 155)**	Oui	2	1,3
	Non	153	98,7

* PCTOMS: Polychimiothérapie OMS

## Discussion

La principale limite de cette étude est liée à son caractère rétrospectif avec son corollaire de dossiers et de données manquantes en raison du mauvais archivage et la non-informatisation des données. Cependant, elle ne manque pas d'intérêt d'autant plus qu'elle porte sur près de deux décennies d'activités de prise en charge des patients atteints de la lèpre dans le district sanitaire de Daoukro. Complétant l'analyse des données de surveillance épidémiologique de routine, elle permettra de mieux cibler les objectifs à atteindre en matière de prévention, de dépistage, de diagnostic et de traitement de la lèpre.

L’évolution de la lèpre de 1999 à 2017 dans le district sanitaire de Daoukro montre 2 phases: 1 phase rapide marquée par une augmentation de l'incidence des cas entre 1999 et 2003, et 1 phase lente de régression de l'incidence entre 2004 et 2017. Depuis 2004, l'incidence est maintenue en dessous de 1 cas pour 10 000 habitants. L'augmentation de l'incidence des cas entre 1999 et 2003 pourrait s'expliquer par l'intensification du dépistage actif par une « équipe mobile » appuyée par une information des populations des districts encore endémiques. Le taux de détection est variable selon les années. Une étude portant sur 5 ans de notification des cas de lèpre dans le district sanitaire de Yamoussoukro, au centre de la Côte d'Ivoire, observait une variation du taux de détection passant de 1,47 pour 10 000 habitants en 1995 à 0,75 pour 10 000 en 1999 avec un maximum de 1,65 pour 10 000 habitants en 1997 [5]. Un regard sur la lèpre au plan mondial permet de noter une baisse générale du nombre de nouveaux cas. En Côte d'Ivoire, les progrès réalisés dans la lutte contre la maladie ont abouti en 2001 à l'atteinte du seuil de l’élimination de la lèpre en tant que problème de santé publique. L'extension des services du programme de lutte contre la lèpre aux zones les plus difficiles à couvrir, l'intensification de la détection active des cas, la formation des agents et la généralisation de la polychimiothérapie ont contribué à l'atteinte de l’élimination de cette maladie [9].

La détection de nouveaux cas chaque année de 1999 à 2015 témoigne d'une transmission non interrompue de l'agent pathogène. Selon Portaels et de Jong [14], cette transmission active est due à plusieurs facteurs tels que la perte d'expertise et d'intérêt vis-à-vis de la lèpre, le dépistage tardif des cas, une transmission à partir de personnes infectées asymptomatiques. L'absence de cas déclaré en 2016 et 2017 doit amener l’équipe cadre du district sanitaire de Daoukro à renforcer la surveillance épidémiologique tant en milieu hospitalier qu'en communauté afin de confirmer ou non l'interruption de la transmission de la maladie. Il importe de souligner qu'en Côte d'Ivoire, deux districts sanitaires sont encore endémiques (Man et Zouan-Hounien) et que l'incidence annuelle nationale est de 0,2 pour 10 000 habitants [9]. Le pays enregistre en moyenne 800 nouveaux cas de lèpre chaque année depuis 10 ans, avec 9% d'enfants, 40% de femmes et 18% d'infirmité de degré 2. Dans notre étude, en 18 années de notification, 185 cas de lèpre ont été déclarés, parmi lesquels les enfants de moins de 15 ans représentaient 16% des cas, les formes multibacillaires 82%, et 24% de cas d'invalidité de degré 2. Une étude au Sénégal, dans la région de Thiès, a permis d'observer dans une cohorte de 63 cas, un effectif de 39 enfants âgés de moins de 15 ans soit 62% [3]. Cette forte proportion d'enfants de moins de 15 ans est explicable par les activités d'une organisation non gouvernementale allemande DAHW (Deutsche Lepra und Tuberkulosehilfe) qui, dans le cadre de la promotion des activités d’élimination de la lèpre au sein des communautés, a mis en place une stratégie de dépistage des enfants à travers la formation des enseignants en milieu rural à la reconnaissance des lésions cutanées. En Côte d'Ivoire, les visites médicales systématiques de la population scolaire pourraient servir d'opportunité pour la recherche active de cas chez les enfants. Nos résultats sont proches de ceux de Kra et Yeboué [7], qui observaient dans leur étude 73% de formes multibacillaires et 38% d'infirmités de degré 2. La proportion élevée de formes multibacillaires, potentiellement contagieuses, traduit un retard diagnostic et un manque de sensibilisation des communautés. Les facteurs associés au retard du diagnostic et du traitement sont variés en Côte d'Ivoire [6]: les imaginaires sociaux négatifs associés à la lèpre, la durée d'hospitalisation et du traitement, la politique de dépistage non adaptée au contexte socioculturel et la méconnaissance des symptômes de la maladie par certains patients (et peut-être par certains soignants ?).

La chimiothérapie chez nos patients a permis d'obtenir des résultats satisfaisants. Parmi les patients chez qui l’évolution de la maladie sous traitement a été précisée, la presque totalité (155/156) a été déclarée guérie avec 2 cas de rechute (2/155). Toutefois, parmi ceux-ci, un quart présentaient des séquelles. Une étude réalisée à Madagascar portant sur le parcours avant diagnostic et les conséquences du retard de prise en charge des lépreux suivis dans la Léproserie Fianarantsoa Madagascar a permis d'observer que la majorité des patients (86%, *n* = 37) présentaient au moins une séquelle de la maladie et plus de la moitié (73%; *n* = 31) avaient un score d'invalidité YMP ≥ 3, et que le délai de parcours avant traitement spécifique de plus de 12 mois était significativement lié à un score d'invalidité ≥ 3 (54% *vs* 19%, *p* = 0,005, OR: 8,6 [1,8-40]) [1]. Le dépistage et le traitement précoce dès les premières manifestations contribueraient à réduire significativement le nombre de cas guéris et à limiter le risque de transmission [12].

Les actions du Programme national d’élimination de la lèpre en général, et en particulier dans le district sanitaire, ont permis une baisse significative de la charge de la lèpre au niveau du district de Daoukro. Il est important que les recherches se poursuivent et proposent des approches et solutions novatrices aux problèmes qui font obstacle au dépistage et aux traitements précoces.

## Conclusion

Cette étude a permis de constater une persistance de la transmission de la lèpre avec toutefois une baisse progressive de l'incidence jusqu’à atteindre 0 cas les 2 dernières années d'observation. Ces indicateurs confirment que depuis plus d'une décennie, le district sanitaire de Daoukro ne fait pas partie des districts endémiques de la lèpre en Côte d'Ivoire. Le nombre de cas de guérison avec séquelles souligne la nécessité d'un dépistage et d'un traitement précoces dès les premières atteintes. Des stratégies axées sur une sensibilisation accrue de la population ainsi que sur une politique de dépistage adéquate de cette maladie sont autant de facteurs qui contribueront à l’éradication de la lèpre en Côte d'Ivoire.

## Contribution des auteurs

Ce travail a été réalisé en collaboration avec l'ensemble des auteurs.

EOT a conçu l’étude, géré la recherche documentaire, supervisé les analyses statistiques, rédigé et révisé l'article. YEK a géré les analyses statistiques et révisé le document. FKE a géré les analyses statistiques et révisé le document. DK a révisé le document. MCD et PSS ont participé à la collecte des données. AY a révisé le manuscrit. OTA a supervisé tout le travail. Tous les auteurs ont lu et approuvé le manuscrit final.

## Liens d'intérêts

Les auteurs déclarent ne pas avoir d'intérêt direct ou indirect (financier ou en nature) avec un organisme privé, industriel ou commercial en relation avec le présent article.
